# Barriers to adoption of recent technology in cervical screening

**DOI:** 10.1186/1742-6413-4-16

**Published:** 2007-08-16

**Authors:** Darshana Jhala, Isam Eltoum

**Affiliations:** 1 Department of Pathology, Division of Anatomic Pathology, University of Alabama at Birmingham, USA

## Abstract

The Pap smear is one of the modern success stories in the field of preventive medicine. Since its introduction as a screening test, there has been a dramatic reduction in the incidence of cervical cancer. However, the search for a better screening test continues. The new technologies, including liquid-based cytology (LBC), Human Papilloma Virus (HPV) testing and automated or machine-assisted screening have been introduced. However, there is continuous debate about whether society's limited resources are better spent on reaching the underserved rather than on these technologies. Another question is whether these technologies create yet another kind of disparity in delivering preventive care. For example, despite the wide use of LBC (99% of tests submitted to our laboratory are LBC), conventional Pap smears are still used to screen/follow up some women. It is not clear why some providers continue to prefer conventional smear over LBC and what are the barriers for adopting LBC in cervical cancer screening. We hypothesize the lower cost of conventional compared to LBC Pap testing, patient's lower socio-economic indices, a patient's medical history and provider's subspecialty/training all appear to play a role in the choice of using conventional Pap testing rather than LBC. Unintentionally, this choice results in repeat testing, delayed treatment and potentially higher costs than intended. The ultimate goal of this review article is to understand and explore possible barriers and disparities to adopting new technology in cancer screening.

## Review

Worldwide, cervical cancer remains one of the leading causes of cancer in women [1]. Pap testing is a simple, safe and economic screening test. In the USA, thanks to screening programs which were initiated more than 50 years ago, the incidence of invasive cervical cancer has dramatically declined. In the last two decades, although there has been a reduction of cervical cancer rates, the reduction had been relatively plateaued during the time period of 1981 to 1997 [2]. Lately, new technologies, such as LBC, Human Papilloma Virus (HPV) testing and automated or machine assisted screening, have been introduced into cervical cancer screening. For many reasons including aggressive marketing, these technologies have replaced the apparently less expensive conventional Pap smear. According to the Washington G2 report, this change has dramatically increased the national expenditure from $1.25 billion to 2 billion (60% increase) [3]. There is a continuous debate, however, on the use and potential abuse of the utilization of these technologies, and on the role of aggressive marketing rather than the ultimate evidence based medicine, in implementing these technologies as screening tests and, lastly, on the role of cost as the ultimate barrier to their adoption.

### Evidence of superiority of LBC over Conventional Pap smears

The accuracy of cervical cytology depends on the quality of the specimen, the preparation of the slide material, and the cytologic interpretation. Sampling technique is of utmost importance [4,5]. Errors in sampling and preparation may be the major cause of false-negative or unsatisfactory Pap smear results [6]. Limitations of the conventional Pap smear are the need for rapid fixation, clumping, and the overlapping of cells with variable thicknesses of the smear. Abnormal cells may be obscured by blood, mucus, and other debris, which potentially leads to an increase in false-negative and equivocal (i.e., ASCUS) results. Slide preparation techniques that use a fluid medium (LBC) have been developed to produce thin layer smears to overcome these limitations.

LBC has been shown to improve the detection rates of cervical intraepithelial neoplasia, both high and low grades, and both squamous and glandular lesions [7-11]. It has also been noted that there are improvements in inter-observer variability [12] and a substantial decrease in inadequate or unsatisfactory smears when utilizing LBC [9]. Some reports document a significant reduction in the false negative rates and improved sensitivity with the use of LBC [8,13,14]. Bernstein, et al, also reported improved sample adequacy leading to improved diagnosis with the use of LBC [15].

At face value, LBC is more expensive than conventional preparations. However, the cost effectiveness may be considered in terms of fewer repeat smears and fewer false-negative cases, most likely as a result of improved sampling. The British National Health Service, through its National Institute of Clinical Excellence, suggests that the LBC technology is economically a better model for screening for cervical pathology. In their study, Doyle, et al. [16] showed a greater than 30% improvement in productivity even if only 73% of the workload was converted to LBC. They also documented that this conversion was slow for the first two years of operation, most probably because of the period of adjustment to the new system. During this period, a number of measures were put in place in order to assure quality; such as double screening of all LBC cases. The most consistent improvements shown with LBP in the literature are in the detection of low-grade abnormalities. Williams, et al. [17] reported that the most significant impact of LBC in their laboratory has been on the rate of unsatisfactory smears which fell from 13.9% with conventional smears, to 1.9% with LBC. The most common reason for unsatisfactory smears with conventional Pap smears are due to air-drying artifact, and obscuring inflammation. These problems are very rare or absent with LBC, giving a much lower rate for unsatisfactory smears. The success of LBC is clearly reflected in the clinics remaining less busy with return/repeat business, although handling a similar load of patients. In one study, prior to LBC, almost 25% of referrals (544 women) were for repeated unsatisfactory smears, but this problem has now almost disappeared, with only 11 women (0.5%) referred in 2003–2004 for this reason [17]. The absolute number of smears reported as showing a glandular abnormality or adenocarcinoma showed a decrease from 66 in 2001–2002 to 45 in 2003–2004. Thus, accuracy is higher, as similar numbers of lesions have been found with both conventional smears and LBC, with fewer glandular abnormality reports with LBC. This may suggest that there has been a lower likelihood of overcalling reactive glandular lesions with LBC.

According to Williams et al, [17] the workload in their laboratory has decreased since the use of LBC. The reasons for this include a decrease in unsatisfactory smears and the fact that LBC smears take less time to screen than conventional smears. Their experience has been that the time taken for a full primary screening has decreased by approximately 40%. There is also an improvement in turnaround times, and backlogs of cases for primary screening have disappeared leading to an improved morale in the laboratory.

### Evidence of a lack of advantage of LBC over conventional Pap smears

Recently the superiority of LBC over conventional Pap smears has been questioned. In a randomized clinical trial from South Africa, Taylor, et al. [18] compared the diagnostic performance of LBC (3184 patients [56.4%]) vs. the diagnostic performance of conventional Pap smears (2463 patients [43.6%]). In contrast to previous findings, they found that the accuracy of both modalities was equivalent – the conventional smear slightly better than LBC. Surprisingly, LBC provided more unsatisfactory samples in comparison to the conventional smears (2.2% vs. 0.8%). (In another publication, Davey, et al. [19] compiled data from 56 studies with a sample size of 1.29 million tests. In their study, the classification of high grade squamous intraepithelial lesion varied according to study quality (p = 0.04) with conventional cytology classifying more slides in this category than did LBC in high quality studies (n = 3) only).

### Current recommendations for the use of the conventional Pap smear

The current recommendations by different national organizations regarding the use of conventional Pap smears or the use of LBC are not entirely clear. In fact, some organizations do not favor or discourage the use of the conventional Pap.

According to the recommendations by the U.S. Preventive Services Task Force (USPSTF) [20], "the evidence is insufficient to recommend for or against the routine use of new technologies to screen for cervical cancer". Although the American Cancer Society has no preference for LBC over conventional Pap tests, it is worth noting that it recommends [21] biennial instead of annual screening if LBC is used instead of conventional Pap smears. ACS suggests that mild abnormalities, seen more frequent with LBC than with conventional smears, lead to unnecessary repeat testing and over-management. The American Society for Colposcopy and Cervical Pathology (ASCCP) guidelines [22] do not have a preference for conventional smears over LBC.

On the other hand, the British National Health Service recommends [23]the use of LBC as the primary means of processing samples for cervical screening in England and Wales. This is based on technical superiority rather than the diagnostic performance of LBC. LBC improves slide preparation, presents a more homogenous sample, and makes the slides easier to read with subsequent increase in productivity. The Danish Center for Evaluation and Health Technology Assessment concludes that from a scientific view either a conventional preparation or a LBC can be chosen [24].

### HPV testing and the HPV vaccine

The cost effectiveness of HPV testing is recognized for patients with a diagnosis of ASCUS. There is also some evidence of the effectiveness of HPV testing and cytology evaluation as primary screening methodology for women >30 years [25,26]. However, whether screening with the combination of HPV testing and cytology for women greater than 30 years will be accepted by women and physicians is unclear [27]. In spite of its effectiveness, the cost of testing may prove to be a barrier for the wide use of HPV testing. Currently, there is no data supporting or refuting this possibility. HPV vaccine is now being considered for girls in the age group of 9 to 13 yrs [28]. It will take some time for implementation of this vaccine. The barriers encountered with vaccines are many and may not be different than barriers encountered when other types of vaccines were first introduced into the marketplace [28-31].

### Automated/Image assisted screening

Automated screening is successful for a wide screen for cervical cancer screening strategies. It has also been shown that automated/image assisted screening is cost-effective, especially in high risk populations [32]. The literature also shows that the use of automated screening reduces the number of false-negative Pap smears [33]. Barriers for the use of automated screening are not well defined, although the initial investment/cost for purchase may be one such factor.

### A System-based approach for assessing possible barriers for adopting recent technologies

It is simplistic to assume that a single factor could explain the preference of one screening method over another. In evaluating barriers to preventive clinical care, Walch and McPhee proposed an innovative model [34] to analyze the different factors that influence the patient and physician choice of preventive measure/behavior such as the Pap test. This model borrows from communication, behavioral, health education and psychological theories and simplifies the analysis of patient-physician interaction.

**Figure 1:**
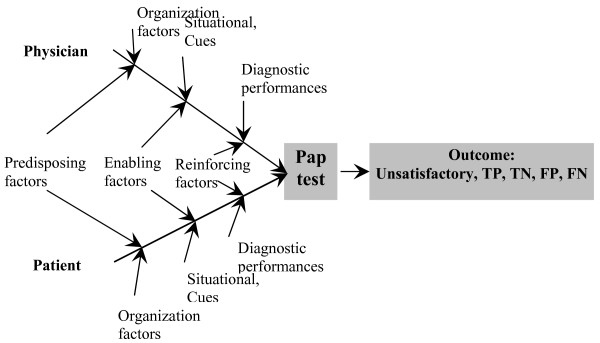
A cause and effect (fish-bone) diagram representing Walsh-McPhee Systems Model for Preventive Care.

This is an innovative empirical model which assumed that the duty of obtaining the recommended preventive care such as a Pap test is not only the responsibility of the women, but a collective result of many interactive factors including those that are related to the individual woman, to the physician counseling the patient, to the healthcare organization (usual place of care), and to the diagnostic performance as well as the cost-effectiveness of the test itself (Figure 1).

The immediate result of this interaction may be the performance of a Pap test with subsequent detection and treatment of intraepithelial lesions – an act which ultimately leads to the reduction of the incidence of cervical cancer. As suggested by Walsh and McPhee [34], the different factors that result in successful screening could be conceptually viewed as a system or as "a cause and effect – fishbone diagram" in which these factors interact to produce the ultimate outcome [34]. For instance, choosing LBC over the conventional method for a Pap test may be associated with decreased numbers of inadequate Pap smears and a better yield for the diagnosis of dysplasia leading to better cytology/histology correlation and avoidance in the delay in the diagnosis of lesions, which leads to better management.

Patient predisposing factors include age, race, education and income. Physician predisposing factors include age, years since graduation from medical school and specialty. Enabling factors include the woman's resources necessary to facilitate screening, for example, the level of insurance coverage. Organizational factors include the health delivery system, office vs. hospital etc. Situational factors/cues to trigger action for cervical cancer screening behavior pertaining to the method of selection for a Pap test include health status and the numbers of visits to physician. Prevention behavior includes having a physician recommendation for screening and prior utilization. Reinforcing factors include geographic area because practice patterns, insurance and HMO penetration, can influence screening and the method selected.

The advantages of Walsh and McPhee [34] model of assessing choice are numerous and include a focus on patient-physician interactions and their unique behavioral factors. The model does not ignore the health care delivery system; neither does it ignore the cost, efficacy and efficiency of the Pap test as a screening tool. Finally, the model approach for identification of barriers in a systemic and systematic way making it easy to investigate.

### Patient barriers

Many patient barriers to cervical screening in general have been described previously [35] including ethnicity (e.g. African -American), inability to speak/understand the English language, no health insurance or being self insured, an unfinished high school education, fatalistic attitudes (including belief that cancer is bad luck, not wanting to know if one has cancer), lack of family support, unable to drive, lower socio-economic status etc., including the inability to afford co-pays. However, a relationship of these factors to the use of conventional Pap smears or LBC has not been explored.

### Provider barriers

Controversial recommendations as discussed above regarding the use of the different technology used for cervical screening, inability to locate the medical records in terms of deciding on a schedule for a regular follow up for screening, the level of knowledge about different techniques used for cervical screening, the education/specialization of a provider are the barriers which may impact cervical screening.

### System barriers

Sometimes the health care system is not well-organized so that it can facilitate screening. Therefore, the educational workshop which focuses on different technologies for cervical screening including advantages and disadvantages may help patients as well as providers in selecting the preferred method of choice.

### The University of Alabama at Birmingham experience

The cytopathology laboratory at the University of Alabama Medical Center is an American-Society-of-Cytopathology designated Center of Excellence. Approximately 250 physicians from more than seven health organizations/settings submit approximately 20,000 Pap tests annually to this laboratory. These organizations included University Hospital, physicians' offices, the Birmingham Veterans Administration Medical Center, Children's Hospital of Alabama, and a large outreach programs around North Central Alabama, including the greater metropolitan areas of Birmingham and Huntsville, Alabama.

The laboratory is manpowered by six cytopathologists (with 8–28 year experience), five cytotechnologists (5–28 years experience), and three medical laboratory assistants (with 2–5 years of experience). All cytologic and histologic data is kept in the Cerner Laboratory Information System^®^. This data is used for clinical, cytological and histological correlation including quality control monitoring which is a continuous process as required by various accreditation agencies.

**Figure 2:**
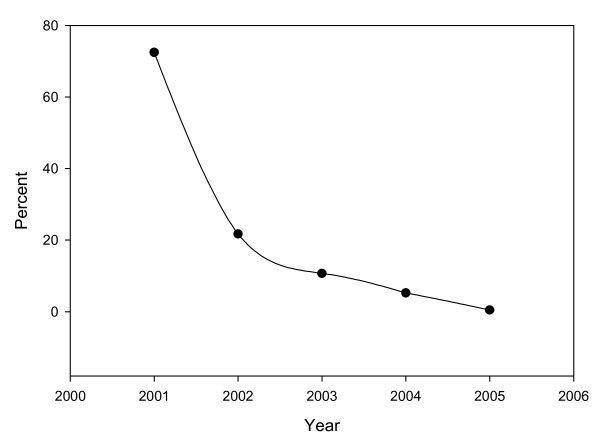
Decrease in the fraction of conventional Pap smear submitted to the Cytology Laboratory, University of Alabama Medical Center, in the last Five years.

Since 1997 when UAB was selected as one of the four national centers for the ALTS trial, the cytology laboratory has utilized LBC. After the issuance of the new Bethesda System and the new guidelines for management of cervical intraepithelial lesions in 2001, the laboratory witnessed a gradual replacement of conventional Pap smear with ThinPrep^® ^LBC (Figure 2).

**Figure 3:**
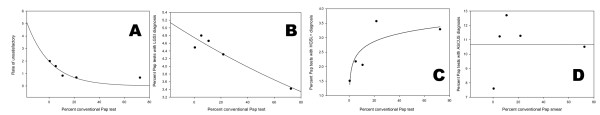
Changes in rates of diagnostic category in relation to the fraction of conventional Pap smear submitted to the Cytology Laboratory, University of Alabama Medical Center. **a**. Correlation between the unsatisfactory Pap test rate and the percentage of conventional Pap smear received in the last 5 years. **b**. Correlation between LGSIL rate and the percentage of conventional Pap smear received in the last 5 years. **c**. Correlation between HGSIL+ rate and the percentage of conventional Pap smear received in the last 5 years. **d**. Correlation between ASCUS rage and the percentage of conventional Pap smear received in the last 5 years.

Currently, 99% of Pap tests are LBC and the remaining 1% are conventional Pap tests. During conversion to ThinPrep^® ^we noticed a statistically significant increase in unsatisfactory specimens and low grade squamous intraepithelial lesions (LGSIL) (Chi-square for trend, P < 0.01). This was collaborated by significant decrease in high grade squamous intraepithelial lesions (HGSIL) rates (Chi Square for trends, P < 0.01). On the other hand, the rate atypical squamous cells of undetermined significance (ASCUS) remained the same (Figure 3).

The changes in these rates may be explained by the conversion to LBC. However, we cannot exclude other reasons such as a change in population demographic and/or a learning-curve effect associated with the adoption of the new Bethesda System or the new guidelines for management of cervical intraepithelial lesions. In the study period, we did not have a change in manpower in the laboratory to explain these observed trends in diagnostic categories.

**Figure 4:**
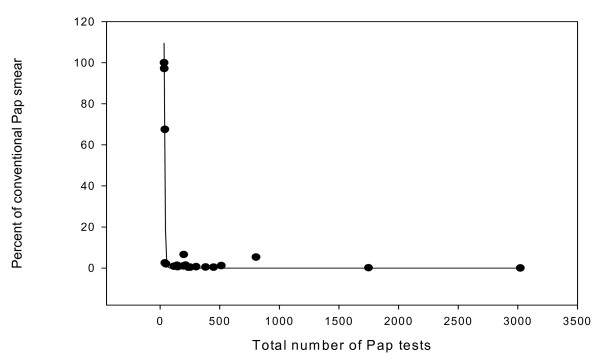
Correlation between the total percent of conventional Pap smear and the total Pap tests performed.

To investigate these observations further, we retrieved all conventional Pap smear reports issued during the period (04/05–04/06) and we determined who ordered the Pap test and from where was it ordered (place of care: VA, Children Hospital, Ob/Gyn service, Hem/Onc service, Primary Care, and others). Additionally, we compared the diagnostic categories for conventional Pap tests to the diagnostic category for LBC for each of these services. Last year, we received 228 conventional Pap smear requests/orders compared to 20,163 LBC, showing that the practicing pattern has been converted dramatically to the use of LBC. Of these, 43 smears came from the VA, 67 smears came from Radiation Oncologists, 27 from Rehabilitation and Physical Medicine, and 13 from Children's Hospital. Very few came from specialized services. The proportion of Pap tests submitted as conventional tests has seen to be inversely related to the total number of Pap test (Figure 4), further suggesting that experience with the Pap test is likely lead to adoption of new technology.

In conclusion, we suggest that the use of Walsh and McPhee [34] Systems Model of Clinical Preventive Care to determine patient and provider barriers for using LBC in cervical screening [34] is useful. Although, socio-economic barriers are explored in the literature in relation to the cervical screening in general, the factors which may affect for the patients as well as providers for the use of recent techniques to obtain Pap test has not been explored. Exploration of these factors, in turn, will help in better deliverance of successful prevention program for cervical screening.
